# Effects of Acute Microcurrent Electrical Stimulation on Muscle Function and Subsequent Recovery Strategy

**DOI:** 10.3390/ijerph18094597

**Published:** 2021-04-26

**Authors:** Alessandro Piras, Lorenzo Zini, Aurelio Trofè, Francesco Campa, Milena Raffi

**Affiliations:** 1 Department of Biomedical and Neuromotor Sciences, University of Bologna, 40127 Bologna, Italy; alessandro.piras3@unibo.it; 2 Department for Life Quality Studies, University of Bologna, 47921 Rimini, Italy; lorenzo.zini2@studio.unibo.it (L.Z.); aurelio.trofe2@unibo.it (A.T.); milena.raffi@unibo.it (M.R.)

**Keywords:** MENS, oxygen consumption, deoxyhemoglobin kinetics, near-infrared spectroscopy, lactate, cycling

## Abstract

Microcurrent electrical neuromuscular stimulation (MENS) is believed to alter blood flow, increasing cutaneous blood perfusion, with vasodilation and hyperemia. According to these physiological mechanisms, we investigated the short-term effects of MENS on constant-load exercise and the subsequent recovery process. Ten healthy subjects performed, on separate days, constant-load cycling, which was preceded and followed by active or inactive stimulation to the right quadricep. Blood lactate, pulmonary oxygen, and muscle deoxyhemoglobin on-transition kinetics were recorded. Hemodynamic parameters, heart rate variability, and baroreflex sensitivity were collected and used as a tool to investigate the recovery process. Microcurrent stimulation caused a faster deoxyhemoglobin (4.43 ± 0.5 vs. 5.80 ± 0.5 s) and a slower VO_2_ (25.19 ± 2.1 vs. 21.94 ± 1.3 s) on-kinetics during cycling, with higher lactate levels immediately after treatments executed before exercise (1.55 ± 0.1 vs. 1.40 ± 0.1 mmol/L) and after exercise (2.15 ± 0.1 vs. 1.79 ± 0.1 mmol/L). In conclusion, MENS applied before exercise produced an increase in oxygen extraction at muscle microvasculature. In contrast, MENS applied after exercise improved recovery, with the sympathovagal balance shifted toward a state of parasympathetic predominance. MENS also caused higher lactate values, which may be due to the magnitude of the muscular stress by both manual treatment and electrical stimulation than control condition in which the muscle received only a manual treatment.

## 1. Introduction

Microcurrent electrical neuromuscular stimulation (MENS) involves series of stimuli delivered superficially, in the microampere range, through special transducer gloves that allow managing microcurrent signals through manipulation techniques. It is a key component for many medical and sport applications, and it is largely used for rehabilitation, training, and recovery purposes [1].

Nowadays, interest in the use of low-intensity current such as MENS is increasing, as its effects take place at the cell level (protein synthesizing activity; increased ATP generation), with sub-sensory application (i.e., painless), besides the absence of collateral effect, low cost, and easy utilization [2]. The utilization of electric field and currents comparable to different cells results in the stimulation of growth and tissue restoration [3] and diminution of edema [4]. Electric stimulations ranging from 10 to 1000 µA increase ATP levels and protein synthesis of rat skin, without having an impact on DNA metabolism [2]. The consequences on ATP production are described by proton actions [5], whereas the amino acids transport through the cell are facilitated by the alterations of the electrical gradients across the membranes [2]. Throughout stimulation of damaged muscles, MENS manages the modified membrane function by various processes, such as the preservation of intracellular Ca^2+^ homeostasis and with the augmented production of ATP levels [6]. Prior studies demonstrated that muscle damage treatment through microcurrent at low amperage (<500 µA) can decrease the severity of muscle symptoms [7] and a quicker regrowth of atrophied animals leg muscles [8]. In addition, microcurrents tone up the smooth muscles of blood vessels, improve skin turgor and tissue temperature, with an increase in blood flow through area treated [9]. All these characteristics are associated with vasodilation, then stimulating the metabolism of waste and toxins from the blood, therefore increasing healing and decreasing pain [2].

Based on these health-related cellular effects, a combination of stimulation plus exercise might improve exercise performance, and it might also be valuable to accelerate the subsequent recovery, thanks to the increased muscle blood flow that accelerates muscle metabolites removal [10]. One of the most physiological variables used to evaluate the recovery process is the heart rate variability (HRV). At the end of exercise, HRV returns exponentially to control value, and its increment is functionally related to athletes’ training status and to the exercise intensity previously executed. HRV is the tool used to analyze the cardiac autonomic responses in combination with the baroreflex sensitivity (BRS), which is a reflex that adapts the heart period in response to variations in systolic blood pressure. These parameters have been used to evaluate the different adaptations to exercise and the recovery times after exercise [11,12,13,14,15]. Regardless of several research and medical applications, few studies have investigated the MENS effect before or after endurance exercise [7,16]. To date, only one study has investigated MENS effects in combination with aerobic exercise in reducing abdominal fat [16]. Authors found that microcurrent application with a frequency range of 25–50 Hz, combined with aerobic exercise, led to a significant decrease in subcutaneous abdominal fat thickness through the lipolysis stimulation [16]. Furthermore, the majority of studies performed with MENS reported a significant reduction of delayed onset muscle soreness after strength exercise [7,17,18]. In elderly people, Kwon et al. [19] found that MENS, after 40 min of short-term application, has an effect on muscle function, enhancing handgrip strength and single leg heel-rise.

Considering the influence of MENS on microcirculation, vascularization, and cellular energy production described above, and that endurance exercise stimulates the microvascular oxygenation following the onset of contractions [20], it could be interesting to investigate the effect of MENS stimulation on muscle tissue oxygenation and its influence on pulmonary oxygen kinetics during cycling. The rapid increase of the pulmonary oxygen kinetics at the transition between rest and exercise is a determinant of aerobic performance and an indicator of a well-done state of oxidative energetic system activity [21]. Additionally, the faster rise in VO_2_ after the onset of exercise indicates a higher muscle oxygen utilization, which is a characteristic of elite athletes and trained subjects [22].

Until now, to our knowledge, no studies have investigated the effects of MENS at the human muscle tissue level, and more precisely, on factors of endurance capacity that are related to performance, such as faster oxygen kinetics, higher muscle oxygen release, or reduced blood lactate level at higher aerobic intensity. Thus, it is possible to hypothesize that the instantaneous and short-term effects of MENS might enhance the individual’s capabilities on exercise at submaximal intensities and to accelerate the subsequent recovery process. Therefore, the aim of our study was to evaluate the acute effects of MENS on the muscle endurance capacity and subsequent recovery in sport. The results could be of great importance for elucidation of the O_2_ release to acute, localized MENS exposure and the development of efficacious performance and recovery modalities.

## 2. Materials and Methods

### 2.1. Participants and Inclusion Criteria

Experiments were performed in 10 healthy subjects (2 females, 8 males; mean ± SD: age 27.2 ± 3.6 years; body mass index (BMI) 23.4 ± 2.5 Kg/m^2^; VO_2_peak 49.9 ± 7.9 mL/kg/min). The subjects were recreationally active but not highly trained. All subjects were volunteers, healthy, non-smokers, and none of them were taking medications or supplements. None of the subjects reported physical deficit or injuries during the study. All participants received a verbal explanation of experimental procedures, and informed consent was obtained before the beginning of recordings. In agreement with the Declaration of Helsinki, the experimental protocol was approved by our University Institutional Ethic Committee.

### 2.2. Study Design and Test Protocol

The study design was a cross-sectional, single-blind, randomized controlled trial. For the realization of this study, the participants visited our laboratory five times, with at least three days between each visit, in which we performed different recordings. In the first visit, the participants performed an incremental test on a cycle-ergometer (H-300-R Lode), to determine ventilatory threshold (VT), respiratory compensation point (RCP), and peak oxygen consumption (VO_2_peak) to identify their individual workload for the succeeding four recording sessions. Expirated gases were analyzed using a Quark b^2^ breath-by-breath metabolic system (Cosmed, Rome, Italy). After the incremental session, the subjects came to our laboratory and performed two repetitions of each conditions of ON (MENS stimulation) and OFF (sham stimulation). The cycling exercise protocol consisted of 1 min of unloaded exercise followed by 5-min of heavy-intensity exercise. On the following days, the four recordings were performed in random order, and the participants were never informed about the status of stimulation, because it was not perceived by the subjects at the cutaneous level (single-blind).

The incremental test consisted of one minute of unloaded pedaling, followed by a warm-up of 5 min at 50 W. Then, at a constant cycling frequency of 75 rpm, the power output started at 80 W and was increased of 20 W/min until volitional exhaustion was reached or the required pedal rate could not be maintained [23]. Ventilatory and gas exchange variables were measured continuously breath by breath throughout the test. The highest VO_2_ averaged over a 20 s interval was taken as VO_2_peak. The LT and RCP were estimated from gas exchange measurements using the V-slope method, ventilatory equivalents, and end-tidal gas tensions [24]. Briefly, VT was determined from a different measurement, such as (i) the first unbalanced increase in CO_2_ production (VCO_2_) with respect to VO_2_; (ii) an increase in expired ventilation (VE/VO_2_) with no increase in VE/VCO_2_; and (iii) an increase in end-tidal oxygen tension with no fall in end-tidal carbon dioxide tension. RCP was determined from a number of measurements including (i) an increase in VE/VCO_2_; and (ii) an increase in end-tidal CO_2_ tension.

**Figure 1:**
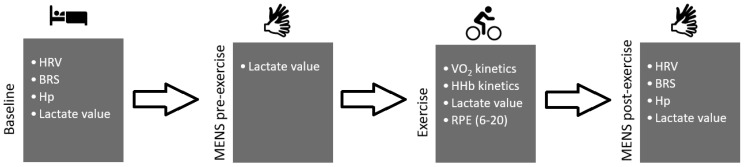
Graphical overview of the experimental protocol. HRV = heart rate variability, BRS = baroreflex sensitivity, Hp = hemodynamic parameters, MENS = microcurrent electrical neuromuscular stimulation, RPE = rate of perceived exertion, VO_2_ = oxygen consumption, HHb = deoxyhemoglobin value. Gloves black and white represent active (ON) and inactive (OFF) stimulation, respectively.

Then, participants come to our laboratory for the exercise sessions. The procedure followed 4 stages: baseline; MENS-pre-exercise; exercise; and MENS-post-exercise, as illustrated below (Figure 1).

*Baseline.* Participants stayed in a supine position in a quiet room, with a comfortable temperature (22–25 °C), for 10 min. They underwent noninvasive continuous blood pressure monitoring using servo-controlled infrared finger plethysmography (Portapres device; TNO/BMI, Amsterdam, the Netherlands) for analysis of heart rate variability (HRV) and baroreflex sensitivity (BRS). HRV is the amount of heart rate fluctuations around the mean heart rate, and it reflects the cardiorespiratory control system. The BRS is an established tool for the assessment of the sympathetic and parasympathetic role of the autonomic nervous system [25]. Tests were performed under a standardized procedure at the same time of the day (9:00–12:00) to prevent circadian effects. Then, we took a blood sample at ear lobe for lactate measurement (Lactate Scout, SensLab, Leipzig, Germany). The reliability of the portable blood lactate analyzer was <0.5 mM for concentrations in the range of ≈1.0–10 mM [26].

*MENS pre-exercise*. Participants, in the same supine position, after the baseline procedure and before exercise, were manipulated with MENS (Electra Microlab, LED, Via Selciatella, Italy). The operator, one of the authors, applied electric pulses through the machine using special transducer gloves to manage microcurrent signals over the most common manipulation techniques, which is necessary to massage and stimulate the quadriceps of the right leg. During sham stimulation (MENS OFF), the operation followed the identical procedure used during the real stimulation, except for the fact that the instrument was turned off. It was possible because the electric pulse was not perceived by the subjects at the cutaneous level (single-blind). The entire massage and stimulation had a duration of 20 min with a Faradic current with rectangular waveform (1 s of impulse duration; frequency of 256 Hz; amplitude of 400 µA; Positive/Negative polarity with change direction), with the intention to stimulate hyperemic vasodilation. After stimulation, we took a second blood sample for lactate measurement; then, participants were ready for the exercise test.

*Exercise.* The data collected during the incremental test were used to calculate the work rates used during the subsequent constant-load exercise tests. Specifically, the individualized workload for each athlete (mean ± SD: 311.4 ± 70.1 watt) corresponded to ~ ≈50% of the difference between power (watt) reached at VT and at the RCP (≈50% Δ RCP-VT). Pedaling frequency was kept at about 70–80 revolutions/min. On-transitions were from unloaded pedaling to the imposed load, which was attained in about 3 s. Pulmonary ventilation (VE), oxygen consumption (VO_2_), and carbon dioxide output (VCO_2_) were determined with a Quark b^2^ breath-by-breath metabolic system (Cosmed, Rome, Italy) previously calibrated according to the manufacturer’s guidelines (included room air calibration, reference gas calibration, and turbine calibration with a 3-L syringe).

The changes in the vastus lateralis muscle oxygenation were evaluated by near-infrared spectroscopy (NIRS). A portable NIRS single-distance continuous-wave photometer (NIMO, Nirox Srl, Brescia, Italy) was utilized for the present study. In brief, the procedure is based on the changes of oxygen absorption with near infrared light, and it includes an emission probe that emits 3 wavelengths (685, 850, and 980 nm) and a photon detector. The transmitted light was recorded continuously at 40 Hz and utilized to quantity deoxygenated myoglobin and hemoglobin levels [27]. The deoxygenated value is less conditioned by the variations of the blood flow, and it is considered as a measure of fractional oxygen extraction inside the microvascular tissue [28]. After that, we had carefully shaven the skin, we attached the probe on it, covering, about 10–12 cm above the knee joint, the lower extremity of the right leg vastus lateralis muscle [29]. Then, the probe and the skin were wrapped with black cloth to prevent corruption from ambient light.

At the third minute of effort, we took a third blood sample for lactate measurement. At the end of the cycling, rate of perceived exertion was recorded with 6–20 Borg scale. Participants were asked how hard they felt the exercise [30]. The constant load exercise had a duration of about 10 min.

*MENS-post-exercise.* Immediately after exercise, participants were back positioned in a supine position, in the same room used before the exercise. To assess the effect of MENS on recovery, we stimulated the quadriceps of the right leg for 20 min with the same protocol described for MENS-pre-exercise. After that, in order to quantify the recovery level, we took a fourth blood lactate and a second continuous blood pressure monitoring using the same plethysmography used before exercise (Portapres device) for HRV and BRS analysis.

### 2.3. Data Analysis

*VO_2_ and HHb Kinetics*. Breath by breath VO_2_ and muscle oxygenation (from this point forward identified as HHb, expressed in µM) data obtained in the different repetitions of the exercise protocol (ON; OFF) were time aligned, interpolated on a second-by-second basis, and then superimposed for every athlete. Average values (every 1 s) were calculated and utilized for kinetics analysis. Data equivalent to the “cardiodynamic phase”, recorded during the first 20 s of the on-transition were not included from the analysis [31]. To evaluate mathematically the VO_2_ and HHb on-transition kinetics, data were fitted with two-exponential terms (primary and slow component of Equations (1) and (2)):

VO_2_(t) = VO_2_(b) + AP * (1 − e − (t-TDp/τp) (phase 2) (primary component) + As * (1 − e − (t-TDs/τs) (phase 3) (slow component)(1)

and

HHb(t) = HHb(b) + AP * (1 − e − (t-TDp/τp) (phase 2) (primary component) + As * (1 − e − (t-TDs/τs) (phase 3) (slow component).(2)

In Equations (1) and (2), Ap, As, TDp, TDs, and τp and τs denote the amplitude, time delay, and time constant, respectively, of the primary and slow component phases. Equations (1) and (2) were used on the basis of which equations yielded the lowest sum of squared residuals. We calculated also the percent contribution of the slow component with respect to the total amplitude of the response. Moreover, the gain of VO_2_, as the increase in VO_2_ above baseline to the reached steady state, and corrected for individualized workload (WL), was also calculated according to this equation (Equation (3)):

Gain = (VO_2_ [150 s − 180 s] − VO_2_ bas)/WL. (3)

*Heart rate variability.* Time and frequency domain parameters were calculated regarding the HRV task force guidelines [25]. For the time domain, the square root of the mean squared differences of successive R-R intervals (RMSSD), and the standard deviation of successive R-R intervals (SDRR) were examined. Spectral analysis provides two main frequency parts: low frequency (LF) ranging between 0.04 and 0.15 Hz and high frequency (HF) positioned at the breathing frequency of 12 breath/minute. It has been revealed that HF is an index of the vagal tone, whereas LF reflects both sympathetic and vagal activities. Both indices (variables with skewed distributions) were log transformed (Ln). The LF/HF ratio provide quantitative markers of the cardiac sympathetic and the vagal modulation [25].

*Baroreflex sensitivity.* It was evaluated with Beatscope version 1.1 a (TNO/BMI, the Netherlands) with a BRS add-on module based on cross correlation analysis [13,14]. The slope of the regression line between SBP (systolic blood pressure) and R-R interval (all intervals between adjacent QRS complexes resulting from sinus node depolarizations) variations are considered as an index of BRS modulation of HR.

*Hemodynamic parameters.* The pulse contour method of Wesseling (the Modelflow method) was used to evaluate cardiac output (CO), stroke volume (SV), ejection time (EJT), and total peripheral vascular resistance (TPR) from the blood pressure waveform [32].

### 2.4. Statistical Analysis

All data are shown as means ± SD. All dependent parameters (VO_2_; HHb; HRV; BRS; hemodynamic and lactate values) were compared between conditions (ON; OFF) with the paired sample t-test, in which means were considered significantly different at *p* < 0.05. To determine the magnitude of the stimulation effects, effect sizes (Cohen’s *d*) were calculated as the mean difference standardized by the between-subject standard deviation and interpreted according to the thresholds: <0.20; small, >0.20–0.60; moderate, >0.60–1.20; large, >1.20–2.00; very large, >2.00–4.00; extremely large, >4.0 [33]. Data were analyzed with SPSS v22.0 (IBM, New York, NY, USA). Regression analysis was done by the least squared residuals technique. Even though several powerful and dedicated software have been commercialized for regression analysis, we used the Solver add-in bundled of Microsoft Excel [34].

## 3. Results

All participants completed the protocol. Individualized oxygen uptake at maximal and submaximal level, with personalized workload obtained at exhaustion during the incremental exercise are shown in Table 1. VT occurred at 63% of the VO_2_peak and at 56% of the maximum workload; consequently, during constant-load exercise, participants pedaled at a mean workload of ~65% (±5) of their maximum.

**Figure 2:**
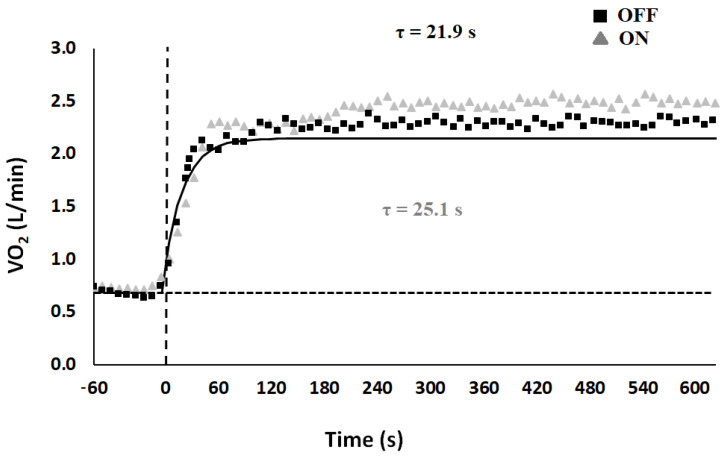
Characteristics of the two-component exponential model describing oxygen uptake (VO_2_) during the on-transient of heavy intensity. Data refer to transition (at time 0, vertical dashed line) from unloaded pedaling to constant-load exercise during OFF (■) and ON (▲) experimental condition. Data points are average values calculated over 1 s. Horizontal dashed line represents baseline. Data obtained during the first 20 s of the transition were excluded from analysis.

*Oxygen uptake kinetics*. Figure 2 shows VO_2_ on-kinetic analysis, in both conditions, for a typical subject. A slow component was observed in both experimental conditions, with non-significant slightly higher value during OFF than the ON condition (4.3% vs. 3.7%). Mean oxygen kinetic parameters for the exponential curve fitting are shown in Table 2. Analysis showed a slower primary component (t(9) = −3.38; *p* = 0.004; mean diff. = 3.25; d = 0.60), with a slower mean response time (t(9) = −2.57; *p* = 0.015; mean diff. = 4.88; d = 0.66) and a shorter time delay of the slow component (t(9) = 1.90; *p* = 0.045; mean diff. = −29.83; d = −0.70) during ON in comparison to the OFF condition. Moreover, VO_2_ at the steady-state level was higher during ON than it was in the OFF condition (t(9) = 2.90; *p* = 0.010; mean diff. = 0.14; d = 0.26). We did not find any significant differences for the increase in VO_2_ per unit increase in work rate (the gain of the primary phase), with values corresponding to 9.14 ± 0.5 and 9.05 ± 0.5 mL/min/W during the OFF and ON condition, respectively.

**Figure 3:**
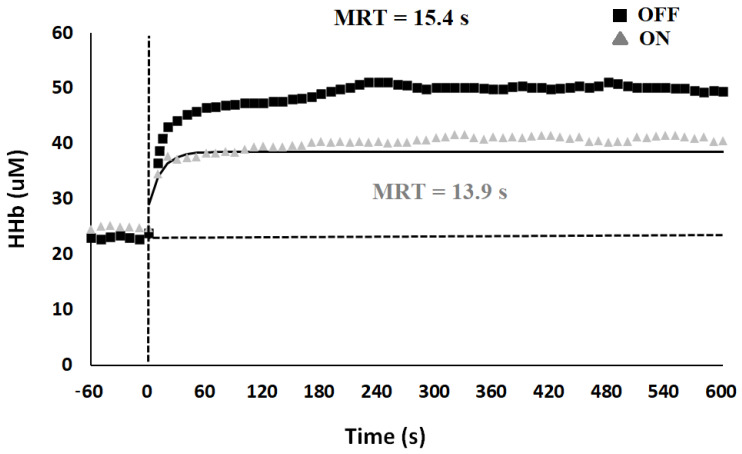
Characteristics of the two-component exponential model describing deoxyhemoglobin (HHb) during the on-transient of heavy intensity. Data refer to transition (at time 0, vertical dashed line) from unloaded pedaling to constant-load exercise during OFF (■) and ON (▲) experimental condition. Data points are average values calculated over 1 s. Data obtained during the first 20 s of the transition were excluded from analysis.

*Muscle oxygenation parameters.* HHb on-kinetics analysis, in both conditions, for a representative subject are shown in Figure 3. Time values of HHb were significantly lower during ON than OFF conditions both at the primary, with faster τp (t(9) = 2.96; *p* = 0.008; mean diff. = −1.37; d = −0.88) and mean response time (t(9) = 2.65; *p* = 0.013; mean diff. = −1.39; d = −0.82), and at the secondary component with faster mean response time (t(9) = 2.35; *p* = 0.022; mean diff. = −32.75; d = −0.63) from rest-to-exercise transition (see Table 3).

*Hemodynamic, cardiac autonomic variables*. Table 4 shows all hemodynamic and autonomic variables investigated. Significant differences were found for systolic (t(9) = 2.67; *p* = 0.013; mean diff. = 7.48; d = 0.70) and mean arterial pressure (t(9) = 2.43; *p* = 0.024; mean diff. = 3.23; d = 0.51), with greater values during ON than OFF conditions. Time and frequency domain analysis showed significant differences for RMSSD (t(9) = 1.75; *p* = 0.047; mean diff. = 4.86; d = 0.28), HF (t(9) = 2.56; *p* = 0.015; mean diff. = 0.30; d = 0.37), and LF/HF ratio (t(9) = 1.95; *p* = 0.044; mean diff. = 0.42; d = 0.62) between ON and OFF conditions, respectively. It appeared that during stimulation (ON), participants recovered faster than during placebo condition (OFF).

**Figure 4:**
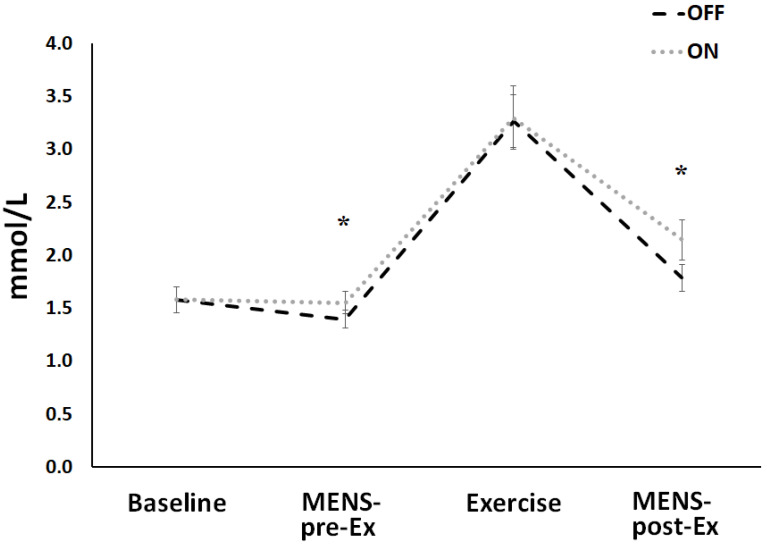
Mean (±SD) lactate values recorded at the baseline, after the first MENS treatment (MENS-pre-Ex) before exercise, at the third minute of the constant-load exercise (Exercise), and after the second MENS treatment (MENS-post-Ex), subsequently to exercise performance. Black dashed line represents OFF, the gray dashed line represents ON condition. Asterisks showed mean significant differences at *p* < 0.05.

*Lactate and the rate of perceived exertion*. Figure 4 shows lactate trends, analyzed at baseline; after the first MENS treatment that preceded the exercise; during the third minute of exercise; and after the second MENS treatment that has followed the exercise. A T-test revealed significant differences for comparison between ON and OFF conditions done before (t(9) = 1.65; *p* = 0.048; mean diff. = 0.16; d = 0.51), and after (t(9) = 1.89; *p* = 0.046; mean diff. = 0.36; d = 0.70) cycling. Rate of perceived exertion showed not significant difference between conditions (12.45 ± 2 vs. 11.25 ± 1.8 for ON and OFF, respectively).

## 4. Discussion

Different studies have discovered that the application of electric fields through the human body can significantly enhance cell metabolism [35] and injury restoration [36] when applied following exercise. The rationale behind the application of MENS is based on its efficacy to generate ATP at the cellular level and other health-related benefits, such as the increase in mitochondrial numbers [16], protein synthesis [7], and the activation of hormone-sensitive lipase, which increases the lipolysis process [16]. According to these different physiological mechanisms found in MENS treatment, our intention was to investigate the short-term effects of MENS on constant-load exercise at submaximal intensities and to the subsequent recovery process. The key results of our study are that MENS stimulation applied before exercise produced an increase in oxygen extraction at muscle microvasculature, while when applied after exercise, improved recovery through faster parasympathetic reactivation with respect to control condition. Moreover, electrical stimulation caused higher lactate levels, which may be due to the magnitude of the muscular stress by both manual treatment and electrical stimulation with respect to the control condition in which the right quadricep received only a manual treatment.

*VO_2_ and HHb kinetics.* The main finding of the present study was the faster HHb on-transition kinetics during exercise executed after MENS stimulation, and surprisingly, by slower VO_2_ on-transition kinetics. After the onset of exercise, a delay has been reported before an increase in muscle O_2_ consumption [37], suggesting that the activation of mitochondrial respiration does not increase immediately, but rather, it has been delayed relative to the start of exercise. It could be argued that combining MENS with exercise might have been increased vasodilation and stimulated hyperemia, which could have, consequently, released nitric oxide, with the effect of accelerating O_2_ availability at the muscle level. Nitric oxide represents an important component of the metabolic inertia to the VO_2_ kinetics during supra-maximal exercise [38]. The precise mechanism by which nitric oxide contributes to the metabolic inertia at exercise onset is unclear but, in vitro, it has been demonstrated its role in inhibiting several mitochondrial enzymes, as it is a competitive inhibitor of oxygen consumption in the mitochondrial respiratory chain [39]. MENS treatment has received more widespread attention in the last years, as it not only relieves pain but also has a positive effect on reparative processes in the skin [1]. Microcurrents penetrate in the body’s cells, normalize the biochemical processes, such as improving metabolism, increasing enzyme activity, ATP synthesis, proteins, lipids, and other vital substances [2]. In addition, microcurrents tone up the smooth muscles of blood vessels as well as improve skin turgor and tissue temperature, with an increase in blood flow through area treated [9]. They are associated with vasodilation, then stimulating the metabolism of waste and toxins from the blood, therefore increasing healing and decreasing pain [2]. Vasodilation and hyperemic processes might have stimulated NO release, even if, to the best of our knowledge, studies are lacking to support this hypothesis.

As shown in previous studies [40,41], the NIRS-derived HHb signal provides a continuous, noninvasive measurement of changes in muscle deoxygenation and reflects the balance between local muscle O_2_ delivery and utilization. Our results have shown an immediate increment in muscle fractional O_2_ extraction after a few seconds of delay (≈10 s) following the onset of contraction. The rate of adaptation of muscle deoxygenation was faster than the adaptation of the primary phase of the VO_2_, reflecting an accelerated O_2_ extraction in the active muscle microvasculature as a consequence of microstimulation. Our results are in agreement with other studies that investigate increasing the availability of muscle O_2_; through hyperoxia, adenosine, or drug administration to the O_2_–hemoglobin dissociation curve, which facilitated O_2_ release at the working muscle, the primary component of pulmonary VO_2_ does not accelerates, even during high-intensity exercise [42,43]. This is in accordance with the hypothesis that VO_2_ during the transition from rest-to-exercise is not managed by the rate of adjustment of convective oxygen delivery to the exercising muscles [42]. After the time delay, during the ON condition, HHb increased more rapidly toward a “steady-state” level, suggesting that oxygen delivery in the on-transition was more adequate to meet the metabolic demand of the muscle, thus requiring a rapid increase in O_2_ extraction [41]. The slightly lower but not significantly different muscle HHb value exhibited by MENS stimulation at steady-state level, in concomitant with the significantly higher value of VO_2_ consumption at the same working rate, suggests that our procedure may have improved oxygen availability/distribution within the muscle microvasculature. The cause of the slower phase II of VO_2_ kinetics is unclear, although it is known that this parameter is sensitive to a number of factors, including the high percentage of type II fiber distribution in the working muscles [44]. However, it is difficult to see how MENS stimulation could alter muscle fiber recruitment patterns, although this should not, of course, be excluded yet.

*Autonomic nervous system parameters.* A second purpose of the present investigation was to examine the different physiological recovery responses to MENS exposure after exercise. The common physiological variable used to evaluate recovery time is the heart rate variability. At the end of exercise, HRV returns exponentially to control value, and its increment is functionally related to the athlete’s training status and the exercise intensity previously executed. HRV is the tool used to investigate the cardiac autonomic responses in combination with the baroreflex sensitivity, which is a reflex that adapts the heart period in response to variations in systolic blood pressure. These parameters have been used to evaluate the different adaptations to exercise and the recovery times after exercise [11,12,13,14]. With a transition from exercise to passive recovery, there is a loss of central command and activation of the arterial baroreflex, resulting in a decrease in heart rate toward its pre-exercise level [45]. The vagal system plays a main role in reducing heart rate immediately after the cessation of exercise, and its further decrease is mediated by both the vagal and sympathetic system [13]. In the present study, we found significantly different effects on the autonomous nervous system parameters, with higher increase in vagal reactivation (RMSSD and HF band of the HRV frequency spectrum) after MENS compared to sham-exposure. Moreover, sympathovagal balance, assessed by LF/HF ratio, was shifted toward a state of parasympathetic predominance, revealing a faster recovery after stimulation treatment than in the control condition. A possible explanation of the microcurrent effects on faster recovery after exercise could be related to its effect on muscle metaboreflex. Until now, no study has investigated the effect of MENS on metaboreflex activity. One study found that the transcutaneous electric nerve stimulation, a technique similar to MENS (both are accepted mode of electrotherapy) [1], augments peripheral blood flow by reduction of the muscle metaboreflex, increasing oxygen supply to stimulated muscles, with a decrease in sympathetic activity evaluated with the heart rate variability [46]. These findings support the idea that the acute application of electrotherapy improves sympathovagal balance, which could be linked to an intense peripheral vasodilatation response, contributing to a faster recovery process.

*Lactate and rate of perceived exertion.* Hyperlactatemia is observed during exercise and severe inflammation [47], as well as in muscle cells subjected in vitro to electrical pulse stimulation [48]. In the present study, lactate levels were significantly higher after MENS treatments, both before and after exercise, whereas during constant-load cycling, participants produced the same lactate values in both experimental conditions. We can speculate that higher lactate values could have caused vasodilation at muscle level, through the changes in osmolarity and acidity, which are necessary to speed-up HHb on-transition kinetics but not higher enough to accelerate VO_2_ on-kinetics. The finding that the lactate values increased with respect to inactive stimulation is somewhat surprising. We could assume that MENS increased the magnitude of muscular activity, which may be due to both manual and electrical stimulation with respect to a sham condition in which the right quadricep received only a manual treatment. Moreover, the lactate is crucial for muscle to make cytosolic NAD+, which is necessary to ATP regeneration from glycolysis, protecting muscles from acidosis. Lactate utilizes two protons, which is necessary to promoting proton elimination from muscles. Moreover, MENS efficacy on blood lactate values could be influenced by parameters used (pulse duration, frequency, amplitude, and muscles stimulated) [1,9], target population [49], and the type of fatiguing exercise or duration of recovery [9,10]. Finally, the RPE was not significantly different between conditions, and this result is similar to that of Barcala-Furelos et al. [10], in which electrical stimulation did not alter the RPE values when compared with the passive recovery in lifeguards following a water rescue.

*Limitations of the study.* Some limitations to the current investigation warrant discussion. Although we know that NIRS has several limitations, most of them have been prevailed by recent technological developments. For example, when the probe is applied on the skin overlying the muscle that we want to investigate, NIRS can measure only a relatively small and superficial volume of skeletal muscle tissue. However, the method has also important strengths and can give valuable and noninvasive useful insights into skeletal muscle oxidative metabolism in vivo during exercise [50].

Furthermore, the main limitation of the whole-body pulmonary oxygen consumption measurements is the difficulty to differentiate between the exercising muscles and the rest of the body, or between different muscles involved in the exercise. Moreover, the presence of O_2_ stores between the location of measurement (the mouth) and the sites of gas exchange at the skeletal muscle level complicate data interpretation during metabolic transitions [50].

The sample size was in line with the most important articles published in this area, in which the number of participants is under 10 units and unbalanced between male and female. Although we have applied it on athletes, it could be important to direct future studies on patients and aged, in which their agility and life quality are limited for impairment in oxygen delivery and utilization.

We have also highlighted the fact that exercise duration, rate of increased in work rate, blood sampling location, instrument utilized, and measurement error are all potential sources of variability in measuring lactate values. However, the reliability of our portable blood lactate analyzer was <0.5 mM for concentrations in the range of ≈1.0–10 mM, with a measurement error of ≤3% [26]. Further investigations are needed, in terms of stimulation parameters (e.g., time, frequency, amplitude, duration) and with different exercise protocols.

## 5. Conclusions

In summary, we found that MENS stimulation causes a faster HHb and a slower VO_2_ on-transition kinetics during exercise, with higher lactate levels immediately after the treatments. Sympathovagal balance was shifted toward a state of parasympathetic predominance, revealing a faster recovery after stimulation executed following cycling. These results could be due to the increased vasodilation and hyperemia, which are a consequence of stimulation. It seems plausible to consider MENS as an electrotherapy useful for improving recovery through faster parasympathetic reactivation following exercise. The absence of any positive effect on VO_2_ on-transition kinetics could be partly imputed to methodological procedures such as the arbitrary choice of stimulation intensity and duration. Nevertheless, additional studies are needed to approve or discard this hypothesis and to shed light on the correlation of these consequences with a short period of training program with concurrent MENS stimulation.

## Figures and Tables

**Table 1:** Oxygen uptake and workload characteristics.

Athletes	VO_2_peak (mL/kg/min)	RCP (mL/kg/min)	VT (mL/kg/min)	VO_2_peak (Watt)	RCP (Watt)	VT (Watt)	Δ50% RCP + VT (Watt)
1	38.63	33.68	25.57	200.00	160.00	114.00	137.00
2	49.66	39.99	33.75	223.00	160.00	139.00	150.00
3	47.08	37.91	26.74	300.00	220.00	126.00	173.00
4	55.56	43.44	34.08	380.00	279.00	218.00	248.50
5	41.60	26.65	21.46	280.00	170.00	130.00	150.00
6	44.68	37.60	24.90	340.00	260.00	162.00	211.00
7	61.97	47.10	42.91	405.00	305.00	274.00	290.00
8	47.46	37.09	30.10	257.00	193.00	140.00	166.50
9	50.61	39.04	32.83	360.00	253.00	220.00	236.50
10	61.75	48.84	41.64	369.00	274.00	220.00	247.00
							
Mean	49.90	39.13	31.40	311.40	227.40	174.30	200.95
SD	7.80	6.40	7.10	70.10	53.90	54.30	52.60
SEM	2.50	2.00	2.20	22.20	17.10	17.20	16.60

Abbreviations: VO_2_peak, peak oxygen consumption; RCP, respiratory compensation point; VT; ventilatory threshold.

**Table 2:** Pulmonary VO_2_ on-kinetics parameters from unloaded pedaling to constant-load exercise across conditions (OFF; ON).

	VO_2_(b) (L/min)	VO_2_(ss) (L/min)	Ap (L/min)	TDp (s)	τp (s)	As (L/min)	TDs (s)	τs (s)	MRTp (s)	MRTs (s)	Sc (L/min)
*OFF*	0.70 ± 0.10	**2.44 ± 0.20**	1.80 ± 0.10	17.58 ± 0.90	**21.94 ± 1.30**	0.20 ± 0.04	**171.70 ± 11.30**	140.85 ± 19.40	**39.52 ± 1.80**	312.55 ± 27.03	0.12 ± 0.02
*ON*	0.79 ± 0.05	2.58 ± 0.20 *	1.79 ± 0.10	19.21 ± 0.90	25.19 ± 2.10 *	0.19 ± 0.04	141.87 ± 15.50 *	159.61 ± 26.10	44.40 ± 2.80 *	301.48 ± 25.90	0.10 ± 0.03

Values are mean ± SD. VO_2_(b), oxygen consumption at baseline level; VO_2_(ss), oxygen consumption at steady-state level; Ap, amplitude of response for primary component; TDp, time delay for primary component; τp, time constant for primary component; As, amplitude of response for slow component; TDs, time delay for slow component; τs, time constant for slow component; MRTp and MRTs, mean reaction time for primary and slow component; Sc, slow component. Bold values with asterisk indicate significant differences between conditions at *p* < 0.05.

**Table 3:** Deoxygenated hemoglobin on-kinetics parameters from unloaded pedaling to constant-load exercise across conditions (OFF; ON).

	HHb(b) (µM)	HHb(ss) (µM)	Ap (µM)	TDp (s)	Τp (s)	As (µM)	TDs (s)	Τs (s)	MRTp (s)	MRTs (s)	Sc (µM)
*OFF*	23.99 ± 3.50	50.40 ± 6.10	24.54 ± 4.10	9.56 ± 0.20	**5.80 ± 0.50**	9.96 ± 2.20	113.22 ± 16.40	110.69 ± 17.60	**15.36 ± 0.50**	**223.91 ± 20.40**	2.47 ± 0.40
*ON*	23.95 ± 3.30	43.08 ± 5.90	19.13 ± 2.80	9.54 ± 0.20	4.43 ± 0.50 *	9.34 ± 2.20	90.61 ± 2.60	100.54 ± 11.70	13.97 ± 0.50 *	191.15 ± 11.40 *	2.11 ± 0.60

Values are mean ± SD. HHb(b), deoxyhemoglobin at baseline level; HHb(ss), deoxyhemoglobin at steady-state level; Ap, amplitude of response for primary component; TDp, time delay for primary component; τp, time constant for primary component; As, amplitude of response for slow component; TDs, time delay for slow component; τs, time constant for slow component; MRTp and MRTs, mean reaction time for primary and slow component; Sc, slow component. Bold values with asterisk indicate significant differences between conditions at *p* < 0.05.

**Table 4:** Hemodynamic and autonomic variables.

	*OFF*	*ON*
**ΔSAP (mmHg)**	**−4.98 ± 2.10**	2.50 ± 2.00 *
**ΔDAP (mmHg)**	0.01 ± 1.50	2.72 ± 2.10
**ΔMAP (mmHg)**	**−1.35 ± 1.50**	1.88 ± 2.00 *
		
**ΔCO (L/min)**	0.16 ± 0.20	0.31 ± 0.30
**ΔSV (mL/min)**	−8.35 ± 1.60	−5.41 ± 3.40
**ΔHR (beat/min)**	7.12 ± 1.70	7.00 ± 1.50
**ΔEJT (s)**	−0.02 ± 0.01	−0.02 ± 0.01
**ΔTPR (mmHg s/mL)**	−0.04 ± 0.05	−0.03 ± 0.10
		
**ΔHRV (ms)**	−140.30 ± 30.10	−132.25 ± 28.80
**ΔSDRR (ms)**	−4.32 ± 4.10	−1.60 ± 4.20
**ΔRMSSD (ms)**	**−12.97 ± 5.80**	−8.10 ± 5.20 *
**ΔLF (Ln/ms^2^)**	0.02 ± 0.20	0.07 ± 0.30
**ΔHF (Ln/ms^2^)**	**−0.43 ± 0.20**	−0.13 ± 0.20 *
**ΔLF/HF**	**0.59 ± 0.20**	0.17 ± 0.10 *
		
**ΔBRS (ms/mmHg)**	−2.75 ± 1.20	−2.15 ± 1.80

Delta values (mean ± SD) are obtained subtracting the recovery values from the baseline values. SAP, systolic arterial pressure; DAP, diastolic arterial pressure; MAP, mean arterial pressure; CO, cardiac output; SV, stroke volume; HR, heart rate; EJT, ejection time; TPR, total peripheral resistance; HRV, heart rate variability; SDRR standard deviation of the R-R intervals; RMSSD, root mean square of the successive differences; LF, low frequency; HF, high frequency; BRS, baroreflex sensitivity; Ln, logarithm. Bold values with asterisk represent significant differences at *p* < 0.05.

## Data Availability

Data sharing is not applicable to this article because of the consent provided by participants on the use of confidential data.
